# Sac-1004, a vascular leakage blocker, reduces cerebral ischemia—reperfusion injury by suppressing blood–brain barrier disruption and inflammation

**DOI:** 10.1186/s12974-017-0897-3

**Published:** 2017-06-23

**Authors:** Haiying Zhang, Joon Ha Park, Sony Maharjan, Jeong Ae Park, Kyu-Sung Choi, Hyojin Park, Yoonjeong Jeong, Ji Hyeon Ahn, In Hye Kim, Jae-Chul Lee, Jeong Hwi Cho, In-Kyu Lee, Choong Hyun Lee, In Koo Hwang, Young-Myeong Kim, Young-Ger Suh, Moo-Ho Won, Young-Guen Kwon

**Affiliations:** 1 Department of Biochemistry, College of Life Science and Biotechnology, Yonsei University, Seoul, 120-749 South Korea; 2 Department of Biomedical Science and Research Institute for Bioscience and Biotechnology, Hallym University, Chuncheon, 24252 South Korea; 3 Department of Neurobiology, School of Medicine, Kangwon National University, Chuncheon, 24341 South Korea; 4 Department of Internal Medicine, School of Medicine, Kyungpook National University, Daegu, 700-721 South Korea; 5 Department of Pharmacy, College of Pharmacy, Dankook University, Cheonan, 31116 South Korea; 6 Department of Anatomy and Cell Biology, College of Veterinary Medicine, and Research Institute for Veterinary Science, Seoul National University, Seoul, 08826 South Korea; 7 Vascular System Research Center, Kangwon National University, Chuncheon, Kangwon 24341 Republic of Korea; 8 Colleges of Pharmacy, Seoul National University, Seoul, 151-742 Korea

**Keywords:** Sac-1004, Cerebral ischemia, Blood–brain barrier, Tight junction, Inflammation, Neuroprotection

## Abstract

**Background:**

Blood–brain barrier (BBB) breakdown and inflammation are critical events in ischemic stroke, contributing to aggravated brain damage. The BBB mainly consists of microvascular endothelial cells sealed by tight junctions to protect the brain from blood-borne substances. Thus, the maintenance of BBB integrity may be a potential target for neuroprotection. Sac-1004, a pseudo-sugar derivative of cholesterol, enhances the endothelial barrier by the stabilization of the cortical actin ring.

**Results:**

Here, we report on the protective effects of Sac-1004 on cerebral ischemia-reperfusion (I/R) injury. Treatment with Sac-1004 significantly blocked the interleukin-1β-induced monolayer hyperpermeability of human brain microvascular endothelial cells (HBMECs), loss of tight junctions, and formation of actin stress fiber. Sac-1004 suppressed the expression of adhesion molecules, adhesion of U937 cells, and activation of nuclear factor-κB in HBMECs. Using a rat model of transient focal cerebral ischemia, it was shown that Sac-1004 effectively ameliorated neurological deficits and ischemic damage. In addition, Sac-1004 decreased BBB leakage and rescued tight junction-related proteins. Moreover, the staining of CD11b and glial fibrillary acidic protein showed that Sac-1004 inhibited glial activation.

**Conclusions:**

Taken together, these results demonstrate that Sac-1004 has neuroprotective activities through maintaining BBB integrity, suggesting that it is a great therapeutic candidate for stroke.

**Electronic supplementary material:**

The online version of this article (doi:10.1186/s12974-017-0897-3) contains supplementary material, which is available to authorized users.

## Background

Ischemic stroke, or cerebral ischemia, is a destructive cerebrovascular condition that has become the leading cause of mortality and morbidity worldwide [1]. Cerebral ischemia induced by a temporary deficiency of blood supply to the brain is known to cause irreversible neuronal death in certain brain regions such as the striatum, neocortex, and hippocampus, which can cause progressive dementia and global cognitive deterioration [2, 3]. Until now, many researchers have attempted to find neuroprotective agents that target pathophysiological mechanisms including inflammation, oxidative stress, apoptosis, and blood–brain barrier (BBB) disruption [4]. Many neuroprotective agents as prospective treatments for ischemic stroke have shown promise in both in vitro and in vivo models of cerebral ischemia; however, their efficacies in patients have been limited [5]. Therefore, there is an urgent need to develop effective neuroprotective agents for the prevention and treatment of cerebral ischemia.

Inflammation plays a central role in the pathogenesis of cerebral ischemia, and the infiltration of various types of inflammatory cells to ischemic regions exacerbates ischemic brain injury [6–8]. Following cerebral ischemia, the expression of inflammatory cytokines such as interleukin-1 beta (IL-1β), tumor necrosis factor-alpha (TNF-α), and IL-6 are elevated [6, 9, 10]. These cytokines induce high levels of expression of adhesion molecules on endothelial cells and other cells [11], including vascular adhesion molecule-1 (VCAM-1) and intercellular adhesion molecule-1 (ICAM-1). These molecules play vital roles in leukocyte adhesion to endothelial cells, leading to infiltration of leukocytes and other activated inflammatory cells into the brain parenchyma across the BBB [12]. Leukocytes also secrete cytokines that cause further activation of glial cells leading to severe brain damage [13].

The BBB is mainly composed of endothelial cells, basement membrane, astrocyte end-feet, and pericytes [14–17]. Under physiological conditions, it is a highly specialized selective barrier and plays an important role in maintaining proper homeostasis of the brain via the control of entry of unnecessary blood-derived toxic components into the brain parenchyma [14]. Endothelial cells are tightly connected by tight junction proteins, especially occludin, claudin-5, and zonula occludin (ZO), to form the BBB [18]. Claudin-5 and occludin are transmembrane proteins which are important for tight junction integrity, and ZO-1 connects it to actin filaments [19, 20]. The reorganization of actin filaments into the cortical actin ring contributes to stabilization of tight junctions, resulting in barrier integrity. The BBB is disrupted under various pathologic conditions, such as cerebral ischemia, Alzheimer's disease, and multiple sclerosis [21]. Dysfunction of the BBB can cause irreversible neuronal damage and brain dysfunction [22, 23]. It is well known that ischemic stroke and ischemia-reperfusion (I/R) injury treated by thrombolytic therapy lead to the disruption of the BBB through tight junction alterations, which allows the infiltration of peripheral immune cells and toxic molecules into the ischemic brain parenchyma, resulting in the development of ischemic neuronal death [24, 25]. Thus, preventing BBB damage may be a promising therapeutic strategy to attenuate the progression of ischemic brain injury [26–28].

In a previous study, we demonstrated that Sac-1004 enhanced the endothelial barrier by forming cortical actin rings via the cAMP/Rac/cortactin pathway, prevented retinal vascular leakage, and reduced tumor vascular hyperpermeability induced by vascular endothelial growth factor (VEGF), histamine, and thrombin in vitro [29]. In addition, Sac-1004 has been shown to block vascular leakage in some conditions including diabetes and cancer [29–31]. In this study, we examined the protective effects of Sac-1004 on BBB disruption using an in vitro BBB model and a rat model of focal cerebral ischemia, which are known to be suitable for evaluating neuroprotective agents and studying the mechanisms of neuroprotective effects [32].

## Methods

### Drug

Sac-1004 was synthesized as described previously [29]. Briefly, Sac-1004 was synthesized via tetrahydropyran deprotection and subsequent glycosidation with 4, 6-di-O-acetyl-2, 3-didieoxyhex-2-enopyran in the presence of acid (Additional file 1: Figure S1A).

### Cell culture

Human brain microvascular endothelial cells (HBMECs) were purchased from the Applied Cell Biology Research Institute (Kirkland, WA). Cells were grown in 2% gelatin-coated dishes, maintained in endothelial cell basal medium (EBM-2, CC-3156) containing EGM-2-kit (CC-4176) (Clonetics, Lonza Walkersville) and 20% fetal bovine serum, and used at passages 5–10.

### BBB permeability assay in vitro

HBMECs were grown until confluent on the luminal side of filters (0.4-μm pore size; Corning) coated with gelatin in 12-well plates. Cells were serum-starved in endothelial serum-free medium for 2 h and treated with Sac-1004 (10 μg/ml) for 60 min before induction with IL-1β (Peprotech, USA) for 3 h. Transendothelial electrical resistance (TEER) was measured with a Millicell ERS-2 volt/ohm meter (Millipore, Billarica, MA). The TEERs of cell-free gelatin-coated filters were subtracted from the measured TEERs and are given as Ω × cm^2^. Paracellular BBB permeability with TEER measurement was confirmed using fluorescein isothiocyanate FITC-dextran fluorescein. FITC-dextran (1 mg/ml; Sigma) was added to the upper compartment. Absorbance of the lower chamber solution was measured at 492 nm (excitation) and 520 nm (emission) in a FLUOstar Omega microplate reader.

### Immunofluorescence staining of HBMECs

HBMECs were fixed in 4% formaldehyde for 20 min at room temperature and permeabilized in 0.1% Triton X-100 in PBS for 15 min at 4 °C. Cells were incubated with antibodies such as rabbit anti-occludin (1:100, Invitrogen), mouse anti-claudin-5 (1:50, Invitrogen), or rabbit anti-zonula occludens-1 (ZO-1, 1:200, Invitrogen) overnight at 4 °C. The cells were incubated with secondary antibodies conjugated with Alexa Fluor for 1 h at room temperature. Actin filaments were monitored with rhodamine phalloidin (Molecular Probes) for 30 min. Cells were mounted using Dako mounting reagent and were observed using a fluorescence microscope (Zeiss; ×400).

### Luciferase assay

Nuclear factor-κB (NF-κB) luciferase reporter constructs were used as previously described [33]. HBMECs were transfected with NF-κB luciferase reporter constructs and pRL-CMV for normalization using Lipofectamine as per the manufacturer’s instructions (Invitrogen). After 24 h, HBMECs were lysed with passive lysis buffer and luciferase activity was measured using the Dual-Luciferase Reporter Assay System (Promega).

### Quantitative real-time reverse transcription polymerase chain reaction

Total RNA was isolated from HBMECs, and cDNA was synthesized using Moloney murine leukemia virus reverse transcriptase. Quantitative real-time polymerase chain reaction (qRT-PCR) was performed with SYBR Green (Invitrogen) in a Bio-Rad real-time PCR detection system. The primers used were as follows: ICAM-1, 5′-GAGGGACCGAGGTGACAGT-3′ and 5′-GTGACCTCCCCTTGAGTGCT-3′; VCAM-1, 5′-GAAGGTGAGGAGTGAGGGGA-3′ and 5′-TTGTATCTCTGGGGGCAACA-3′; and GAPDH, 5′-CCCTCCAAAATCAAGTGGGG-3′ and 5′-CGCCACAGTTTCCCGGAGGG-3′.

### Monocyte adhesion assay

HBMECs were plated in 96-well plates at 2 × 10^4^ cells/well overnight. U937 cells were labeled with 5 μM calcein-AM (Sigma-Aldrich, St Louis, MO) and incubated at 37 °C for 30 min. Calcein-AM-labeled cells were co-cultured with confluent endothelial cells for 1 h and then washed with PBS to remove unbound cells. Fluorescent intensity was measured by fluorometer at 494 nm (absorbance) and 517 nm (emission) (BGM LABTEC, Offenburg, Germany).

### Triton X-100 fractionation

Triton X-100 fractionation was performed as described previously with minor modifications [34]. HBMECs plated in 60 mm plates were serum-starved for 3 h. They were then pretreated for 1 h with or without Sac-1004 (10 μg/ml) prior to stimulation with IL-1β (10 ng/ml) for 3 h. They were then fractionated in cytoskeleton-stabilizing buffer (10 mM HEPES [pH 7.4], 250 mM sucrose, 150 mM KCl, 1 mM EGTA, 3 mM MgCl_2_, 1 mM Na_3_VO_4_, 0.5% Triton X-100, and 1× protease inhibitor cocktail [Roche Diagnostics Modular, Germany]) by centrifugation at 13000 rpm for 20 min. The proteins in the TritonX-100-insoluble and soluble fractions were analyzed by western blotting.

### Western blot analysis

HBMECs were washed with cold PBS, harvested in cytosolic buffer (10 mM Tris [pH 7.5], 0.05% NP-40, 3 mM MgCl_2_, 100 mM NaCl, 1 mM EGTA, 1 mM Na_3_VO_4_), incubated for 5 min at 4 °C, and centrifuged at 3000 rpm for 5 min. After centrifugation, nuclei were pelleted and suspended in nuclear buffer (1 mM EDTA, 3.5% SDS, 10% glycerol, and 70 mM Tris–Cl), as described previously [35]. The proteins were separated by SDS polyacrylamide gel electrophoresis (PAGE). Immunoblotting was performed with antibodies to NF-κB p65, β-actin (Santa Cruz Biotechnology, Santa Cruz, CA) and proliferating cell nuclear antigen (PCNA, Millipore, Billerica, MA).

### Experimental animals

Male Sprague-Dawley rats (8 weeks of age; body weight, 260–280 g) to be used as an animal model of transient focal cerebral ischemia were purchased from Charles River Laboratories (Seoul, Korea). The animals were housed in a conventional state at an adequate temperature (23 °C) and humidity (60%) with a 12-h light/12-h dark cycle and provided with free access to water and food. The animals were acclimated to their environment for 5 days before being used in the experiments.

### Introduction of transient focal cerebral ischemia

The rats were initially anesthetized with a mixture of 2.5% isoflurane (Baxtor, Deerfield, IL) in 33% oxygen and 67% nitrous oxide via face mask. Anesthesia was maintained with 2% isoflurane. A rectal temperature probe was introduced, and a heating pad maintained the body temperature at 37 °C during the surgery. Focal cerebral ischemia was induced by middle cerebral artery occlusion on the right side as described previously [36]. Briefly, the right common carotid artery was exposed through a midline cervical incision. The right external carotid artery was dissected free and isolated distally by coagulating its branches and placing a distal ligation prior to transection. A piece of 3-0-monofilament nylon suture (Ethicon, Johnson-Johnson, Brussels, Belgium), with its tip rounded by gentle heating and coated with 0.1% (*w*/*v*) poly-l-lysine, was inserted into the lumen of the right external carotid artery stump and gently advanced 18 mm into the internal carotid artery from the bifurcation to occlude the ostium of the middle cerebral artery occlusion. After 2 h of ischemia, the suture was pulled back, and the animals were allowed to recover. Finally, the animals were kept in a thermal incubator (Mirae Medical Industry, Seoul, South Korea) to maintain their body temperature until euthanization. Sham-operated animals were subjected to the same surgical procedures except that the middle cerebral artery was not occluded.

### Treatment with Sac-1004

To elucidate the neuroprotective effect of Sac-1004 on ischemic damage, the experimental animals were randomly divided into three groups: (1) the sham-operated group (sham group), (2) the vehicle-treated ischemia-operated group (vehicle-ischemia group), and (3) the Sac-1004-treated (0.5 mg/kg) ischemia-operated group (Sac-1004-ischemia group). Sac-1004 was dissolved in absolute ethanol and then diluted to the desired concentration with saline (final concentration of ethanol 3%). Sac-1004 was administered intravenously 30 min after ischemic surgery. The vehicle-ischemia group received the same dose of ethanol dissolved in saline.

### Neurological deficits

Neurological scores 1 day after transient focal cerebral ischemia (*n* = 7 per group) were evaluated as described previously [37]. The following scoring was used: 0, no observable neurological deficit; 1, flexion of the contralateral torso and forelimb upon lifting of the whole animal by the tail; 2, circling to the contralateral side when held by the tail with the feet on the floor; 3, spontaneous circling to the contralateral side; and 4, no spontaneous motor activity. All animals’ scores were estimated within approximately 1 min, and estimation was repeated another three times for consistency. A score of 0 corresponded to a normal neurological status and higher scores corresponded to behavioral deficits.

### Positron-emission tomography

After evaluation of neurological deficits, positron-emission tomography (PET) evaluation of brain function (*n* = 7 per group) was carried out and cerebral glucose metabolism was measured to evaluate brain function. The animals were anesthetized with 1.5–2% isoflurane in 33% oxygen and 67% nitrous oxide 20 min before being intravenously injected with 100 μCi of ^18^F-FDG (fluorine-18 fluoro-deoxy-glucose) through the tail vein. The animals were placed in a prone position using a stereotaxic head holding device to improve the accuracy of coregistration, and PET imaging was performed 1 h after FDG injection using a small-animal PET scanner (Inveon PET; Siemens). Images were acquired under inhalation anesthesia (isoflurane, 1.5–2%), and FDG/PET images were reviewed using fusion software (Syngo, Siemens; Knoxville, TN). PET images were displayed in axial, coronal, and sagittal planes, and they were available for review. The level of radioactivity in the brain tissue (percentage dose per gram) was estimated from the images according to the method published by Hsieh [38].

### Measurement of infarct volume

Infarct volume was measured according to our published procedure [36]. At 1 and 4 days after transient focal cerebral ischemia, the animals (*n* = 7 per group) were anesthetized with pentobarbital sodium and sacrificed. Their brains were cut into coronal slices of 2 mm in thickness using a rat brain matrix (Ted Pella, Redding, CA, USA). The brain slices were then incubated in 2% 2,3,5-triphenyltetrazoliumchloride (TTC, Sigma-Aldrich, St. Louis, MO, USA) at 37 °C for 20 min to reveal the ischemic infarction. After the TTC reaction, the cross-sectional area of infarction and non-infarction in each brain slice between the bregma levels of +4 mm (anterior) and −6 mm (posterior) was measured using Image J analysis software (version 1.6 NIH). Unstained areas (pale color) were defined as ischemic lesions. The infarct volume was calculated according to the slice thickness of 2 mm per section. Each side of the brain slices was measured separately, and the mean values were calculated. The total volume of infarction was determined by integrating six chosen sections and is expressed as a percentage of the total brain volume.

### Evaluation of BBB permeability

BBB permeability was assessed by measuring Evans Blue (Sigma-Aldrich, St. Louis, MO, USA) extravasations using the modified method of a previous study [39]. Briefly, Evans Blue dye (2% in 0.9% saline, 2 mL/kg) was injected into the tail vein immediately after I/R. At 3 h after I/R, the animals (*n* = 7 per group) were anesthetized with sodium pentobarbital and transcardially perfused with physiological saline and then decapitated. The brains were removed, and each hemisphere was weighed, homogenized in PBS, and centrifuged at 2000×*g* for 15 min at 4 °C. Then, 0.5 mL of the resulting supernatant was added to an equal volume of trichloroacetic acid. After overnight incubation and centrifugation at 2000×*g* for 15 min at 4 °C, the supernatant was taken for spectrophotometric quantification of extravasated Evans Blue dye at 620 nm. The quantitative calculation of the dye content in the brain was based on external standards dissolved in the same solvent. The results are expressed as micrograms per gram brain tissue.

### Tissue processing for histology

For the histological analysis, the animals were anesthetized with sodium pentobarbital and perfused transcardially with 0.1 M PBS (pH 7.4) followed by 4% paraformaldehyde in 0.1 phosphate buffer (pH 7.4). The brains were removed and fixed in the same fixative for 6 h and cryoprotected by infiltration with 30% sucrose overnight. Thereafter, the brain tissues were serially sectioned on a cryostat (Leica, Wetzlar, Germany) into 30-μm coronal sections.

### Immunohistochemistry for SMI-71 and GLUT-1

To examine changes in SMI-71 (an endothelial barrier antigen) and glucose transporter-1 (GLUT-1; an endothelial cell marker) immunoreactivities in the ischemic cortex (*n* = 7 per group) 3 h after I/R, immunohistochemical staining for mouse anti-SMI-71 (1:500, Covance) or rabbit anti-GLUT-1 (1:1000, Chemicon International) was performed as described previously [40]. In brief, the sections were incubated with diluted mouse anti-SMI-71 or rabbit anti-GLUT-1 overnight at 4 °C. The tissues were then exposed to biotinylated goat anti-mouse IgG or anti-rabbit IgG and streptavidin peroxidase complex (Vector, USA). They were then visualized with 3, 3′-diaminobenzidine in 0.1 M Tris HCl buffer and mounted on gelatin-coated slides. After dehydration, the sections were mounted with Canada balsam (Kato, Japan).

To quantitatively analyze their immunoreactivities, the corresponding areas of the cerebral cortex were measured from eight sections per animal. Images of all SMI-71 and GLUT-1 immunoreactive structures of the cerebral cortex were taken using a light microscope (BX53, Olympus, Germany) equipped with a digital camera (DP72, Olympus) connected to a PC monitor. Images were calibrated into an array of 512 × 512 pixels corresponding to a tissue area of 250 × 250 μm (40 × primary magnification). The densities of all SMI-71 and GLUT-1 immunoreactive structures were evaluated on the basis of optical density (OD), which was obtained after the transformation of the mean gray level using the formula: OD = log (256/mean gray level). After the background was subtracted, a ratio of the OD of the image file was calibrated as a percentage (relative optical density, ROD) using Adobe Photoshop version 8.0 and then analyzed using NIH Image 1.59 software (National Institutes of Health, Bethesda, MD). The mean value of the OD of the sham group was designated as 100%, and the ROD of each group was calibrated and expressed as a percentage of the sham group.

### Immunofluorescence staining

The brain sections were incubated in blocking solution for 2 h at room temperature, and then incubated at 4 °C overnight with one of the following antibodies: mouse anti-CD31 (1:100; BD Pharmingen), rabbit anti-ZO-1 (1:100; Invitrogen), rabbit anti-occludin (1:100; Invitrogen), rabbit anti-claudin-5 (1:50; Invitrogen), mouse anti-glial fibrillary acidic protein (GFAP, 1:1000; Millipore), mouse anti-CD11b (1:100; BD Pharmingen), rabbit anti-VCAM-1 (1:100; Santa Cruz), and rabbit anti-ICAM-1 (1:100; Santa Cruz). After five washes in 0.1% Triton X-100 in PBS for 15 min each, the sections were incubated with secondary antibody overnight at 4 °C. Before washing, the sections were treated with 1 μg/ml 4′,6-diamidino-2-phenylindole (DAPI) and washed five more times with 0.1% Triton X-100 in PBS for 30 min each. All antibodies were dissolved in antibody diluent (Dako). Confocal images were captured at room temperature with ZEN software on an upright confocal microscope (LSM 700; Carl Zeiss) using the predefined ZEN software configurations for Alexa Fluor 546, Alexa Fluor 488, and DAPI.

### Statistical analysis

Data are presented as the means ± standard errors of the mean (SEM). All statistical analyses were performed using GraphPad Prism (version 5.0; GraphPad Software, La Jolla, CA). Differences of the means among the groups were statistically analyzed by two-way analysis of variance (ANOVA) with post hoc Bonferroni’s multiple comparison tests in order to elucidate ischemia-related differences among the experimental groups. *P* < 0.05 was considered to be statistically significant.

## Results

### Sac-1004 blocks IL-1β-induced blood–brain barrier hyperpermeability in HBMECs

**Fig. 1:**
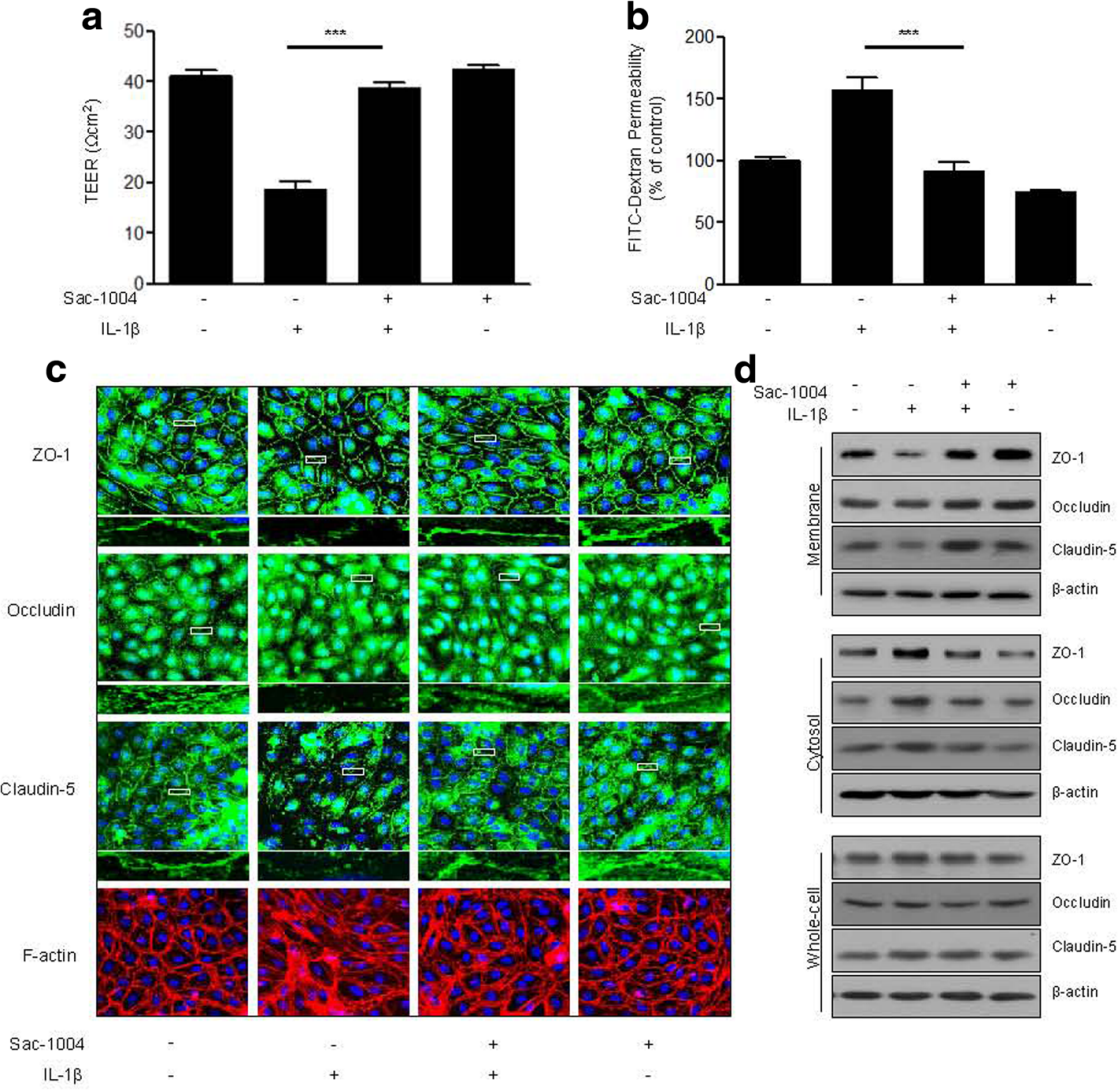
Sac-1004 blocks IL-1β-induced BBB disruption in HBMECs. HBMECs were starved and treated with or without Sac-1004 (10 μg/ml, 1 h) prior to stimulation with IL-1β (10 ng/ml, 3 h). Sac-1004 blocked both the TEER decline (**a**) and the increase in FITC-dextran transendothelial permeability (**b**) induced by IL-1β. TEER was measured using Millicell ERS-2 (Millipore). For the permeability assay, FITC-dextran was added to the upper chamber. Absorbance of the solution in the lower chamber was measured at 492 nm (excitation) and 520 nm (emission) in a FLUOstar Omega microplate reader. HBMECs were starved and treated with or without Sac-1004 (10 μg/ml, 1 h) prior to stimulation with IL-1β (10 ng/ml, 2 h) (**c**). Cells were then fixed, permeabilized, and subsequently immunostained for ZO-1, occludin, claudin-5, and F-actin. *Rectangle*: the region enlarged in high-power images. Translocation of tight junction proteins was assessed as described in the methods section (**d**). Whole-cell lysates, Triton X-100-insoluble and soluble fractions were subjected to SDS-PAGE followed by western blot analysis with anti-ZO-1, anti-occludin, anti-claudin-5 and anti-actin. Blots are representative of three independent experiments. All data are presented as means ± SEM. ***P < 0.001

Numerous studies have shown that IL-1β, which is prominently unregulated in ischemic lesions [9, 41, 42], induces BBB breakdown [43–47]. This can be used to stimulate the BBB and mimic in vitro stroke conditions. To observe the protective effects of Sac-1004 on BBB integrity, we used in vitro models of the BBB involving the mono-culture of HBMECs and stimulated the cells with IL-1β in the presence or absence of Sac-1004 for 3 h. Potential changes in the integrity of the BBB were assessed by measuring TEER and the permeability of the HBMEC monolayer to FITC-dextran [48]. Sac-1004 blocked IL-1β-induced TEER decline and FITC-dextran leakage (Fig. 1a, b). Sac-1004 also increased HBMEC viability under serum-free conditions (Additional file 1: Figure S1B and C).

The major component of the BBB is the tight junction complex, which plays an important role in maintaining BBB integrity and is stabilized by close connection to the actin cytoskeleton. Therefore, we decided to test the effect of Sac-1004 on the stability of the tight junction protein occludin, claudin-5, and ZO-1 expression by immunostaining. Normally, confluent HBMECs display a linear pattern of tight junction proteins at the cell borders and this characteristic localization was disrupted by IL-1β (Fig. 1c). The disruption effects were blocked by Sac-1004. Furthermore, F-actin staining data showed that control confluent HBMECs had ring-like shapes. IL-1β treatment disrupted cortical actin ring structures and increased actin stress fibers. Sac-1004 markedly prevented IL-1β-induced stress fiber formation and maintained the cortical actin ring shape (Fig. 1c).

In order to determine the significance of this finding, we examined the effect of Sac-1004 on occludin, claudin-5, and ZO-1 proteins using a fractionation method. In untreated confluent HBMECs, these proteins were predominantly present in the membrane fraction. Interestingly, we found that IL-1β treatment increased the proportion of tight junction proteins in the cytosolic fraction with a reciprocal decrease in the amount of tight junction proteins in the membrane fraction and that this was restored by treatment with Sac-1004 (Fig. 1d). Their total expression level in the whole-cell lysates remained unchanged (Fig. 1d). Collectively, these results demonstrate that Sac-1004 has a barrier protective effect by stabilizing tight junction proteins and the actin cytoskeleton.

### Sac-1004 inhibits IL-1β-induced expression of adhesion molecules and monocyte adhesion to HMBECs

**Fig. 2:**
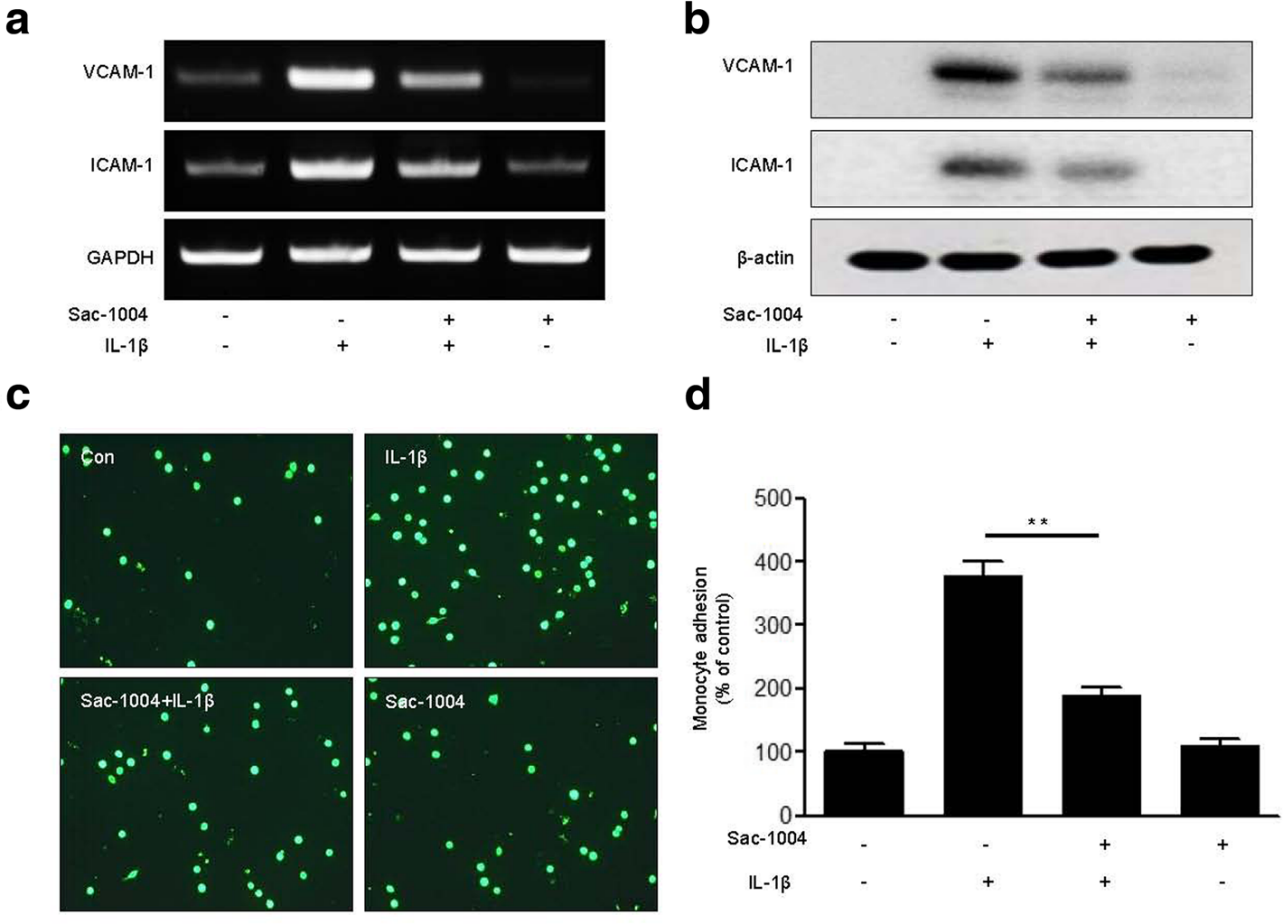
Sac-1004 attenuates IL-1β-induced adhesion of U937 cells to HBMECs. HBMECs were allowed to grow to confluence. Starved cells were treated with Sac-1004 (10 μg/ml, 1 h) followed by IL-1β (10 ng/ml, 6 h). RNA levels of VCAM-1 and ICAM-1 were measured using RT-PCR (**a**). Protein levels were analyzed using the indicated antibodies (**b**). HBMECs were treated with IL-1β (10 ng/ml, 6 h) and co-cultured with calcein-AM-labeled monocytes for 1 h. Representative images show the reduction in the IL-1β-induced adhesion of U937 cells to HBMECs (**c**, **d**). Attached monocytes were imaged by fluorescent microscopy. The fluorescent intensity of attached monocytes was quantified by a fluorometer at 494 nm (absorbance) and 517 nm (emission). All data are presented as means ± SEM. **P < 0.01

Monocyte recruitment, adhesion, and transendothelial migration are key features of the inflammatory response in ischemic stroke [11, 49]. ICAM-1 and VCAM-1 are key elements that mediate the adhesion of leukocytes to the vascular endothelium [50]. We observed that Sac-1004 pretreatment inhibited IL-1β-induced ICAM-1 and VCAM-1 expression at both the mRNA and protein levels (Fig. 2a, b).

Next, we performed a monocyte adhesion assay to investigate the effect of Sac-1004 on monocyte adhesion to HBMECs. Figure 2c shows that treatment of HBMECs with IL-1β significantly increased monocyte adhesion to the endothelial cell monolayer. Sac-1004 attenuated this IL-1β-induced adhesion of monocytes to the endothelial monolayer (Fig. 2c, d). Taken together, Sac-1004 inhibits IL-1β-induced monocyte adhesion to endothelial cells by downregulating adhesion molecule proteins.

### Sac-1004 inhibits IL-1β-induced NF-κB activation in HBMECs

**Fig. 3:**
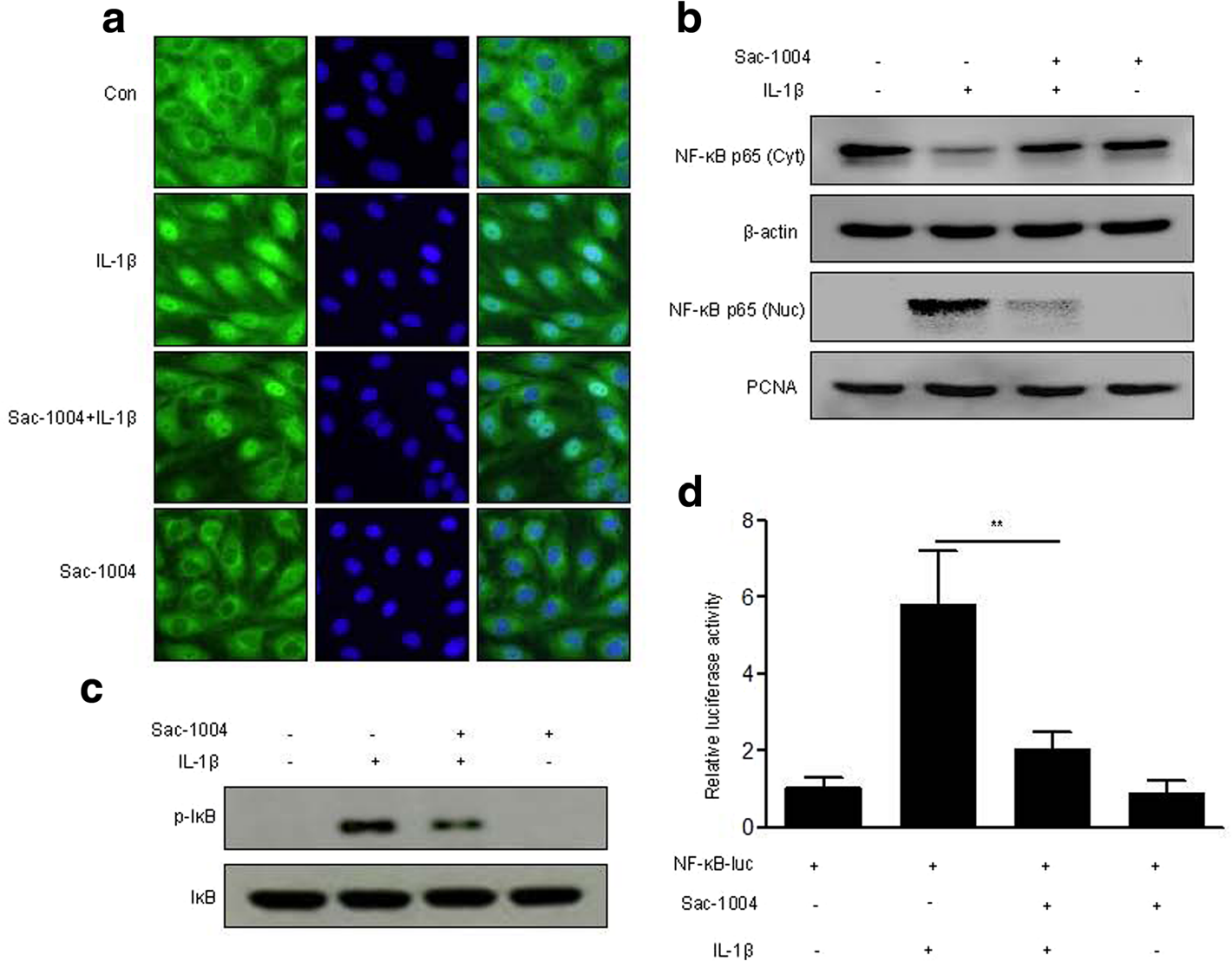
Sac-1004 suppresses IL-1β-induced NF-kB activation. HBMECs were allowed to grow to confluence. Starved cells were treated with Sac-1004 (10 μg/ml, 1 h) followed by IL-1β treatment. Western blotting was performed using anti-p-IκB and anti-NF-κB antibodies. Cells were then fixed, permeabilized, and subsequently immunostained for NF-κB. Translocation of the NF-κB protein to the nucleus and cytosol fractions was observed. IL-1β (10 ng/ml, 6 h) induced the translocation of NF-κB to the nucleus, but in the presence of Sac-1004, the translocation of NF-κB was reduced (**a**, **b**). Sac-1004 reduced the IL-1β (10 μg/ml, 30 min)-induced expression level of p-IκB (**c**). HBMECs were transfected with a NF-κB p65 reporter construct. The next day, the transfected cells were treated with Sac-1004 (10 μg/ml, 1 h) followed by IL-1β (10 ng/ml, 12 h) treatment. Luciferase activity from the NF-κB p65 reporter constructs was measured. The luciferase reporter assay also showed a similar reduction in IL-1β-induced NF-κB activation after treatment with Sac-1004 (**d**). Data are shown as relative activity compared with a mock vector. Transfection efficiency was normalized to Renilla luciferase activity from co-transfected pRL-CMV. All data are presented as means ± SEM. *P < 0.05

NF-κB is a key transcription factor in the regulation of adhesion molecule expression [11]. We measured the levels of the NF-κB p65 subunit in the cytoplasm and in the nucleus of Sac-1004-pretreated cells. Sac-1004 prevented IL-1β-induced p65 translocation into the nucleus (Fig. 3a). This was additionally confirmed by western blotting (Fig. 3b). We also observed that Sac-1004 decreased IL-1β-induced p65 phosphorylation (Fig. 3c). Furthermore, when the cells were pretreated with Sac-1004, IL-1β-induced NF-κB activity was reduced (Fig. 3d). These results indicate that Sac-1004 reduces IL-1β-induced NF-κB activation.

### Sac-1004 attenuates brain damage after I/R

**Fig. 4:**
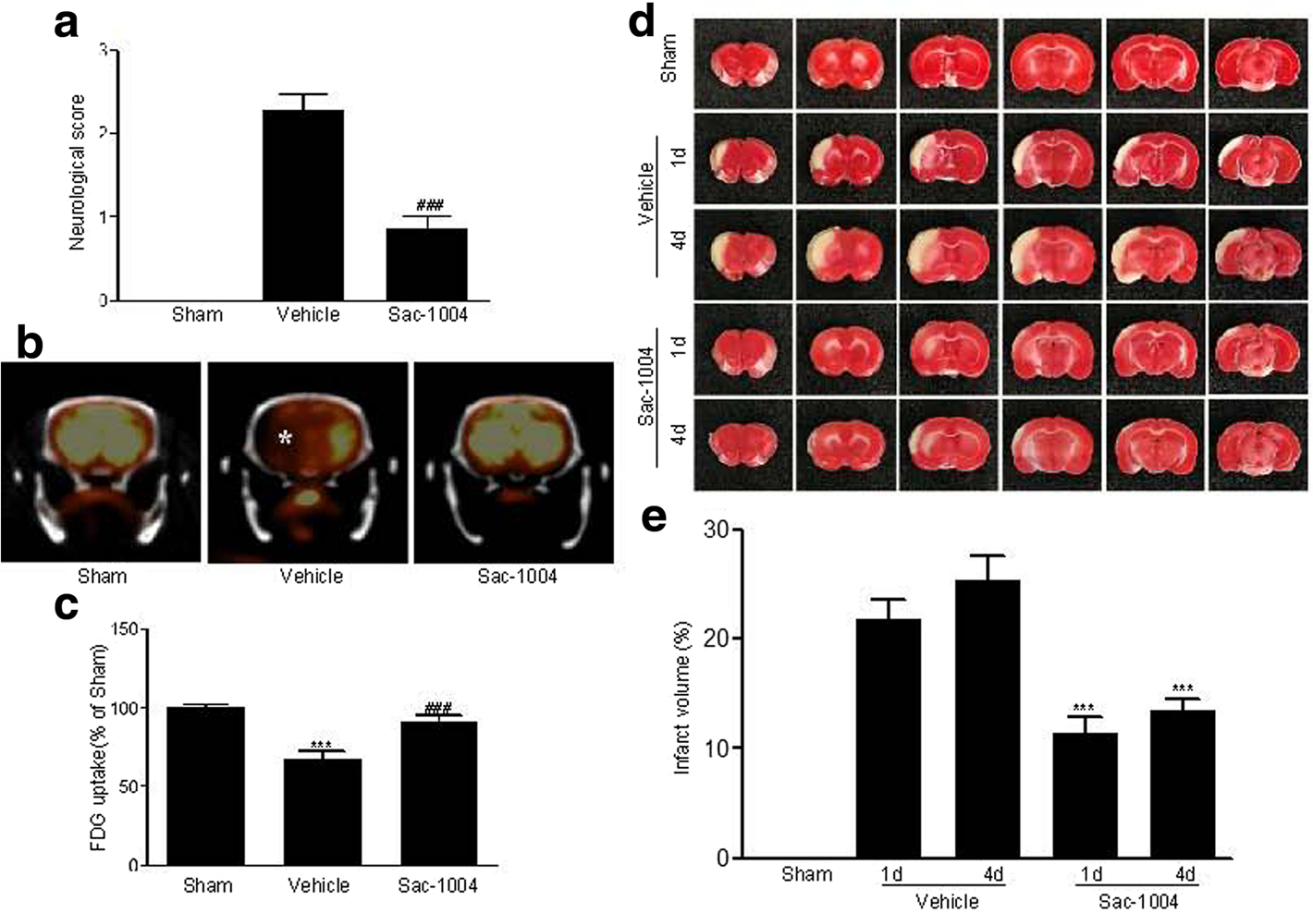
Sac-1004 decreases cerebral injury after I/R. Neurological score (**a**) and PET imaging (**b**) in the sham, vehicle-ischemia, and Sac-1004-ischemia groups 1 day after I/R. Severe neurological deficits were observed, and ^18^F-FDG uptake (*asterisk*) was significantly decreased in the vehicle-ischemia group. However, in the Sac-1004-ischemia group, neurological deficits were significantly reduced and ^18^F-FDG uptake was increased compared with those in the vehicle-ischemia group. **c** Relative analysis as percentage values of ^18^F-FDG uptake groups 1 day after I/R (*n* = 7 per group; ^***^ *P* < 0. vs sham group, ^*###*^ *P* < 0.001 vs vehicle-ischemia group). TTC staining (**d**) in the sham, vehicle-ischemia, and Sac-1004-ischemia groups on days 1 and 4 post ischemia. Severe infarction was easily observed in the vehicle-ischemia group 1 and 4 days after I/R. However, infarct regions were significantly decreased in the Sac-1004-ischemia group. (**e**) Percentage change of infarct volume 1 and 4 days after I/R (*n* = 7 per group; ^***^ *P* < 0.001 vs vehicle-ischemia group). The bars indicate the means ± SEM

To strengthen the in vitro data, we used a rat model of transient focal cerebral ischemia. In order to evaluate neurological impairment, neurological scores were evaluated 1 day after I/R (Fig. 4a). In the vehicle-ischemia group, severe neurological deficits were exhibited compared with the sham group. However, treatment with Sac-1004 significantly prevented the neurological impairments evoked by I/R.

Cerebral glucose metabolism was evaluated via ^18^F-FDG-PET 1 day after I/R (Fig. 4b, c). ^18^F-FDG uptake was distinctly decreased in the vehicle-ischemia group compared with the sham group. However, ^18^F-FDG uptake in the Sac-1004-ischemia group was significantly increased compared with that in the vehicle-ischemia group.

TTC staining was used to examine infarct volume 1 and 4 days after transient focal cerebral ischemia; the pale stained area denoted the infarct area (Fig. 4d, e). In the sham group, no infarction was present in any cerebral regions. In the vehicle-ischemia group, infract regions were easily observed in the cerebral cortex and striatum 1 and 4 days after I/R; there was no significant difference between the infarct volumes on day 1 and day 4 after I/R. However, in the Sac-1004-ischemia group, the infarct volumes were significantly reduced compared with those in the vehicle-ischemia group 1 and 4 days after I/R; although, no significant difference between the infarct volumes on days 1 and 4 post ischemia were observed.

### Sac-1004 reduces BBB leakage after I/R

**Fig. 5:**
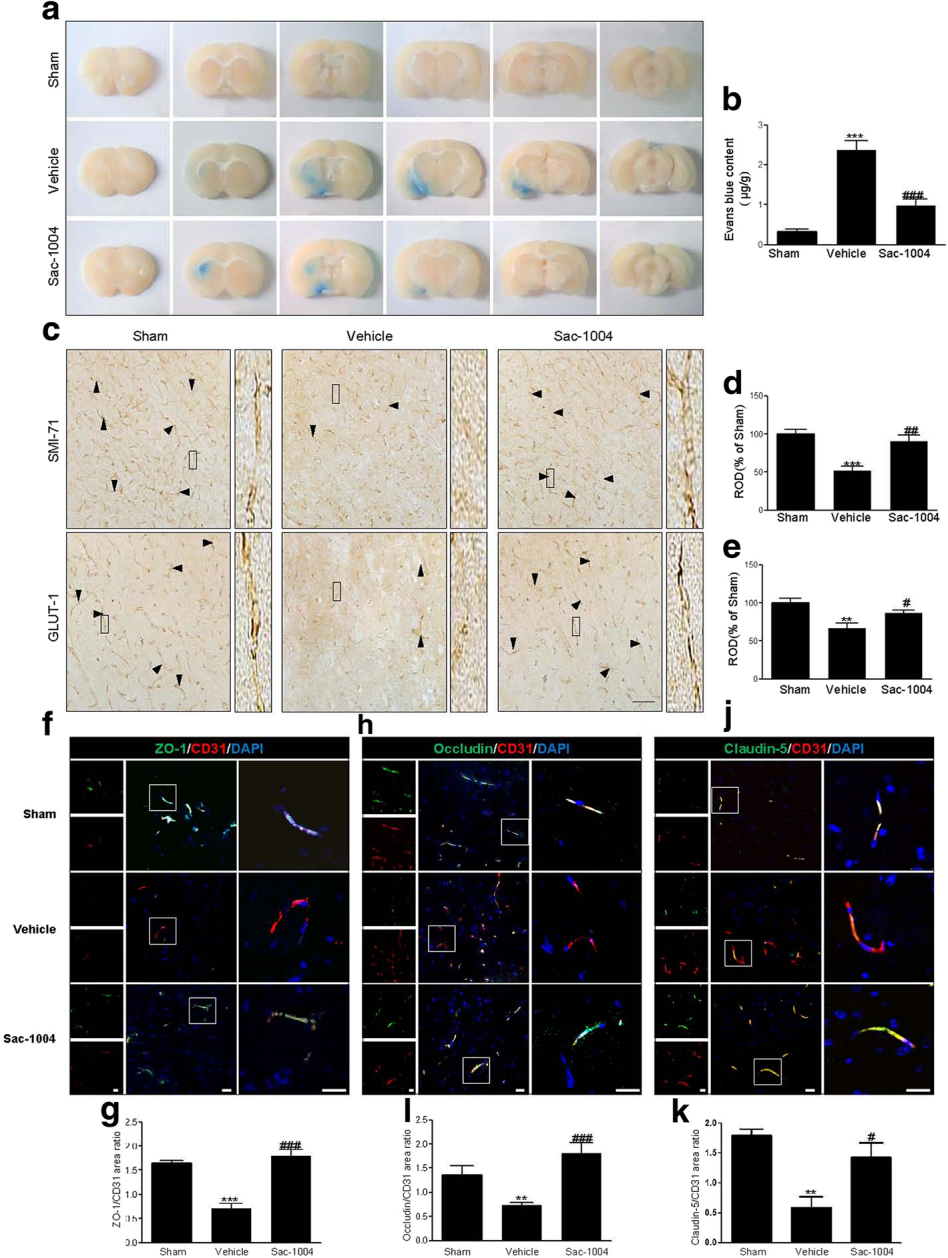
Sac-1004 blocks BBB disruption after I/R.Evans Blue dye extravasation (**a**) in the sham, vehicle-ischemia, and Sac-1004-ischemia groups 3 h after I/R. In the Sac-1004-ischemia group, the amount of Evans Blue dye extravasation was significantly decreased in the ischemic brain compared with that in the vehicle-ischemia group. **b** The quantitative analysis of Evans Blue leakage 3 h after I/R (*n* = 7 per group; ^***^ *P* < 0.001 vs sham group, ^*###*^ *P* < 0.001 vs vehicle-ischemia group). SMI-71 and GLUT-1 immunohistochemistry (**c**) in the ischemic cortex of the sham, vehicle-ischemia, and Sac-1004-ischemia groups 3 h after I/R. In the sham group, SMI-71 and GLUT-1 immunoreactions were easily observed in microvessels (*arrowheads*) in the cerebral cortex, and their immunoreactivities were significantly decreased in the vehicle-ischemia group. However, in the Sac-1004-ischemia group, SMI-71 and GLUT-1 immunoreactivities were significantly higher than those in the vehicle-ischemia group. *Rectangle*: the region enlarged in high-power images. Scale bar = 60 μm. **d** and **e** ROD as percentage values of SMI-71 and GLUT-1 immunoreactivities in the ischemic cortex 3 h after I/R (*n* = 7 per group; ^**^ *P* < 0.01 and ^***^ *P* < 0.001 vs sham group, ^*#*^ *P* < 0.05 and ^*##*^ *P* < 0.01 vs vehicle-ischemia group). **f** Immunofluorescence staining for ZO-1 (*green*) and CD31 (*red*) in the ischemic cortex of the sham, vehicle-ischemia, and Sac-1004-ischemia groups 3 h after I/R. Merged images of ZO-1 and CD31 staining are also shown. *Square*: the region enlarged in high-power images. Scale bar = 20 μm. **g** Quantitative assessment of ZO-1 positive blood vessels. **h** Immunofluorescence staining for occludin (*green*) and CD31 (*red*) in the brain sections. Merged images of occludin and CD31 staining are also shown. *Square*: the region enlarged in high-power images. Scale bar = 20 μm. **i** Quantitative assessment of occludin positive blood vessels (*n* = 5 per group; ^***^ *P* < 0.001 vs sham group, ^*###*^ *P* < 0.001 vs vehicle-ischemia group). **j** Immunofluorescence staining for Claudin-5 (*green*) and CD31 (*red*) in the brain sections. Merged images of claudin-5 and CD31 staining are also shown. *Square*: the region enlarged in high-power images. Scale bar = 20 μm. (**k**) Quantitative assessment of claudin-5 positive blood vessels (*n* = 5 per group; ^**^ *P* < 0.01 vs sham group, ^*#*^ *P* < 0.05 vs vehicle-ischemia group). The *bars* indicate the means ± SEM

The effects of Sac-1004 on BBB permeability 3 h after I/R were evaluated with Evans Blue extravasation (Fig. 5a, b). In the ischemic brain of the vehicle-ischemia group, the amount of Evans Blue dye extravasation was significantly higher than that in the sham group. However, treatment with Sac-1004 significantly decreased ischemia-induced Evans Blue dye extravasation.

To corroborate this result, immunohistochemical staining with SMI-71 and GLUT-1 was performed to examine morphological changes of microvessels in the ischemic cortex 3 h after I/R (Fig. 5c–e). SMI-71 and GLUT-1 immunoreactions were easily observed in the microvessels of the cerebral cortex in the sham group. In the vehicle-ischemia group, SMI-71 and GLUT-1 immunoreactivities were significantly decreased compared with those in the sham group. However, in the Sac-1004-ischemia group, SMI-71 and GLUT-1 immunoreactivities were significantly higher than those in the vehicle-ischemia group.

Furthermore, the tight junction-related proteins occluding, claudin-5 and ZO-1 were examined 3 h after I/R by immunofluorescence microscopy in conjunction with CD31, an endothelial marker. In the sham group, occludin and CD31 signals were aligned almost perfectly, whereas in the vehicle-ischemia group the alignment became disorganized, with the occludin signal being greatly reduced, indicative of a damaged BBB (Fig. 5f). However, in the Sac-1004-ischemia group, a certain degree of rescue of the superimposed lining of occludin and CD31 was observed, suggesting that the BBB destruction after I/R was attenuated (Fig. 5f). Similar results were observed with claudin-5 and ZO-1 (Fig. 5h, i). Together, these results further demonstrate that BBB destruction after I/R injury could be effectively rescued by Sac-1004 treatment via restoring tight junction expression.

### Sac-1004 suppresses expression of adhesion molecules and activation of glial cells after I/R

**Fig. 6:**
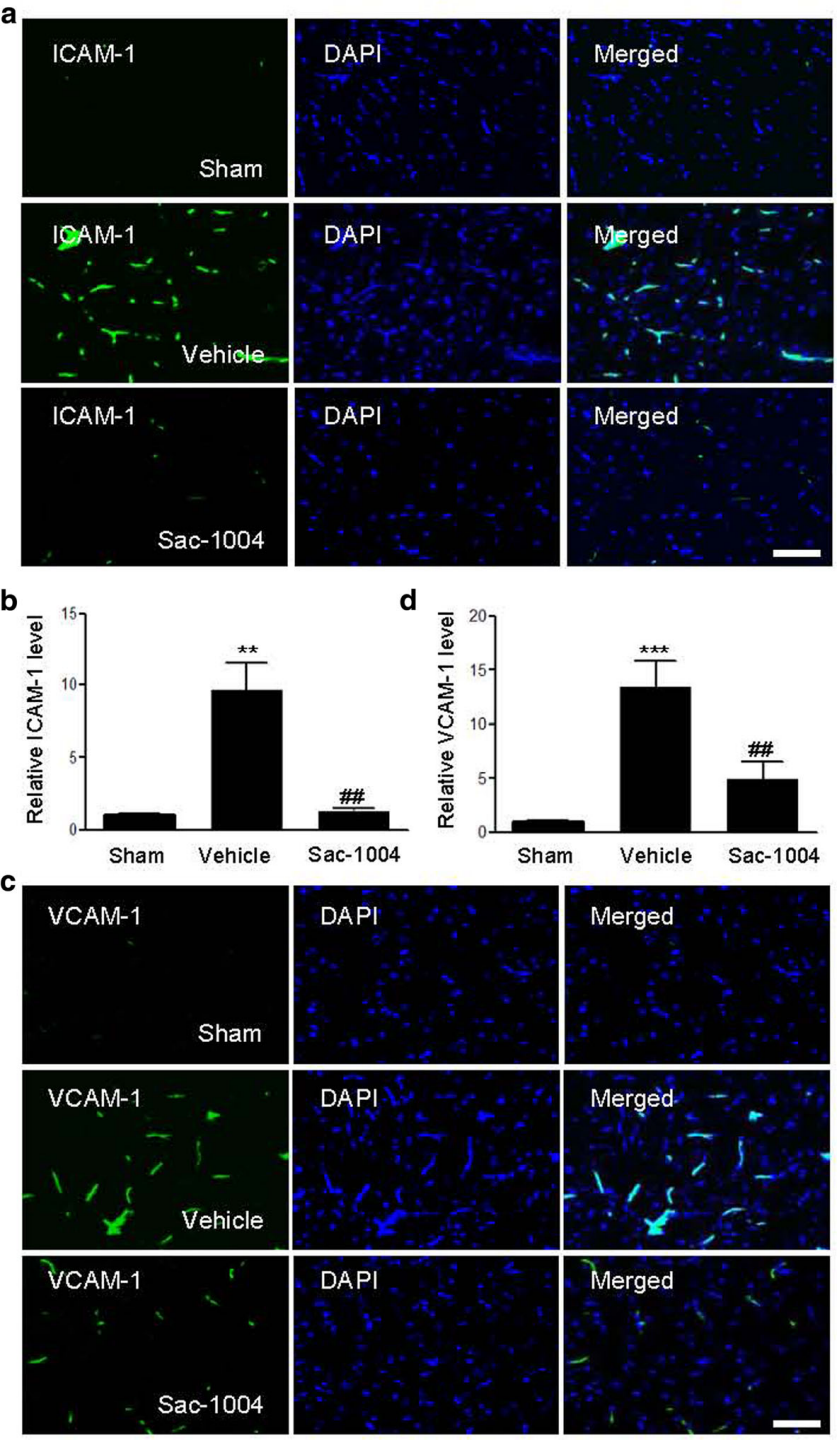
Sac-1004 attenuates expression of adhesion molecules after I/R. **a**, **c** Immunofluorescence staining for ICAM-1 and VCAM-1 in the ischemic cortex of the sham, vehicle-ischemia, and Sac-1004-ischemia groups 3 h after I/R. Merged images of ICAM-1 or VCAM-1 and DAPI staining are also shown. Scale bar = 50 μm. **b**, **d** Quantification was done using Image J. (*n* = 5 per group; ^**^ *P* < 0.01 and ^***^ *P* < 0.001 vs sham group, ^*##*^ *P* < 0.01 vs vehicle-ischemia group). The *bars* indicate the means ± SEM

Both ICAM-1 and VCAM-1 are barely expressed in normal brain cells, but their levels are increased during inflammation following I/R [42]. In immunofluorescence staining, expression levels of ICAM-1 and VCAM-1 were significantly decreased in the microvessels of the Sac-1004-ischemia group 3 h after I/R compared with those in the vehicle-ischemia group (Fig. 6a, b).

**Fig. 7:**
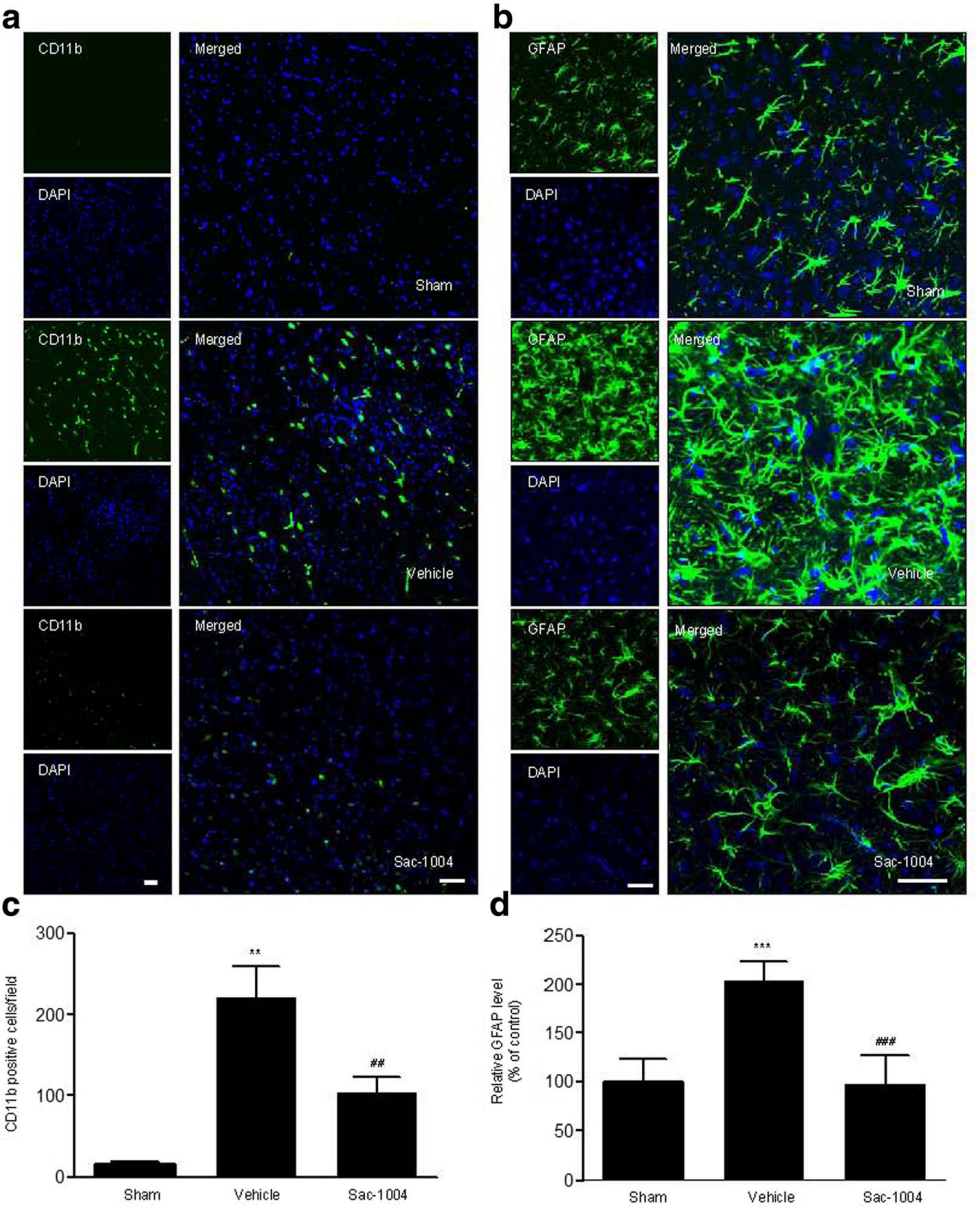
Sac-1004 inhibits glial activation after I/R. Immunofluorescence staining (**a**, **b**) of CD11b and GFAP in the ischemic cortex of the sham, vehicle-ischemia, and Sac-1004-ischemia groups 3 h after I/R. Profound expression of CD11b and GFAP was observed in the vehicle-ischemia group compared with that in the sham group, whereas the Sac-1004-ischemia group showed reduced expression of CD11b and GFAP. Scale bar = 50 μm. Glial activation(**c**, **d**) is quantified by the intensity of CD11b and GFAP immunofluorescence (*n* = 5 per group; ^**^ *P* < 0.01 and ^***^ *P* < 0.001 vs sham group, ^*##*^ *P* < 0.01 and ^*###*^ *P* < 0.001 vs vehicle-ischemia group). The bars indicate the means ± SEM

Activation of glial cells such as astrocytes and microglia is crucial in neuroinflammation induced by cerebral ischemia [51]. To investigate the effect of Sac-1004 on neuroinflammation in the ischemic cerebral cortex 3 h after I/R, we observed the morphological changes of astrocytes and microglia using immunofluorescence staining. Transient focal cerebral ischemia induced the activation of GFAP-positive astrocytes and CD11b-positive microglia. However, injection of Sac-1004 after reperfusion significantly inhibited the activation of microglia and astrocytes (Fig. 7a, b). Statistical analysis of the GFAP and CD11b signals indicated that in the Sac-1004-ischemia group, glial activation was significantly attenuated compared with that in the vehicle-ischemia group. Together, these results demonstrate that Sac-1004 attenuated neuroinflammation by inhibiting glial activation.

## Discussion

Ischemic stroke is often accompanied by BBB disruption, inflammation, and oxidative stress [6, 52, 53]. BBB deficit triggers vascular edema and hemorrhage, creates an inflammatory environment, and finally results in neuronal death and brain damage [17, 54]. Thus, it may be irrefutable to suggest that the maintenance of BBB integrity is a key strategy to protect the brain from I/R-induced injury. We previously developed a vascular leakage blocker, Sac-1004, which promisingly reduced VEGF-mediated endothelial permeability and improved endothelial junction integrity and pathological vessel normalization in diabetic retinopathy and tumor angiogenesis [29, 31, 55]. Here, we extended our previous finding’s results and demonstrated that Sac-1004 inhibited IL-1β-induced endothelial permeability by stabilizing tight junction complexes, attenuated inflammation responses induced by IL-1β through inhibiting NF-κB activation, and significantly decreased neurological deficits, cerebral infarction, and glial activation in a rat model of transient focal cerebral I/R.

In the early period after cerebral I/R injury, pro-inflammatory cytokines such as IL-1β, TNF-α are released by neuronal, glial, and endothelial cells [42], and numerous studies have demonstrated that these factors can contribute to BBB disruption in vivo and in vitro [56–59]. Furthermore, it has been reported that BBB permeability increases in the ischemic brain between 3 and 5 h after I/R injury [60]. The disruption of BBB integrity following cerebral ischemia occurs at an early stage of ischemia damage, which is related to an increase in cerebral blood flow [53]. Thus, an earlier safeguard against pro-inflammatory cytokine-mediated BBB impairment can efficiently protect the brain from I/R injury. Our study showed that Sac-1004 in IL-1β-exposed brain endothelial cells reduced endothelial leakage by analyzing TEER and FITC-dextran permeability and the expression pattern of tight junction proteins. Notably, we found that BBB integrity in I/R injury was also preserved by Sac-1004-induced stabilization of tight junction proteins and SMI-71 and GLUT-1. A decreased and dysregulated expression pattern of these proteins can be highly susceptible to brain damage during ischemic events [61–63]. The Sac-1004-mediated restoration of these proteins can be an important step to preserve an intact brain region from ischemic damage. Endothelial junction stability by Sac-1004 would have caused enhanced pericyte recruitment, and this efficiently resulted in increased vascular normalization, as suggested in our previous report on tumor vessels [31]. It was supported by CD31 immunoreactivity pattern that morphology of abnormal vessels after I/R injury were normalized by Sac-1004 treatment. Normalization with Sac-1004 is likely to result in the reduction of infarct size and brain edema after I/R injury. Besides, our unpublished data show that Sac-1004 decreased neuronal death in an animal model of transient global cerebral ischemia, in part, that Sac-1004-mediated BBB stabilization could lead to the mitigation of neural damage. Collectively, our study results have clearly shown that cerebral vascular integrity is a crucial factor to improve brain damage and that Sac-1004 is an efficient vascular leakage blocker to protect the brain from threatening conditions such as ischemic damage.

Together with BBB leakage, brain inflammation occurs in the endovascular area and parenchyma of the ischemic brain and is likely to be related to the activation of endothelial and glial cells [64, 65]. During inflammation, a complex network of cytokines and dysregulated adhesion molecules provoke the recruitment and invasion of leukocytes, which contribute to the exacerbation of brain injury [66]. Adhesion molecules facilitate the adhesion of leukocytes to endothelial cells. The activation of the NF-κB pathway, which is commonly used as an indicator of inflammation in cerebral ischemia studies, is well known to mediate the expression of adhesion molecules [67]. After stimulation, IκB proteins are phosphorylated and degraded, allowing the translocation of the p65 component of NF-κB to the nucleus, followed by the activation of specific target genes such as adhesion molecules [42]. In the present study, we observed that Sac-1004 decreased monocyte adhesion not only to IL-1β-mediated brain microvascular cells but also to brain cells after I/R injury. Its detailed mechanism showed that these effects of Sac-1004 are likely attributed to the prevention of the translocation of p65 into the nucleus and phosphorylation of IκB, suggesting that Sac-1004 exerts anti-inflammatory effects through the NF-κB pathway. Besides, Sac-1004 alleviated glial activation after I/R injury. Numerous studies have suggested that glial activation is involved in both neuronal cell death and endothelial impairment [68]. In particular, the glia is a component of the BBB structure and contributes to BBB integrity and function [69–71]. As dysregulated glia cause impairment in brain function by resulting in endothelial and neuronal damage, the regulation of glia activity by Sac-1004 likely contributes to alleviate the extent of cerebral infarction and neuronal deficiency. Taken together, our study results suggest that Sac-1004 rescues endothelial and neuronal cells from ischemic inflammatory damage, even though further investigations are required to clarify these issues.

Ischemic stroke is a devastating condition; the only current U.S. Food and Drug Administration-approved ischemic stroke therapy is thrombolysis by treatment with tissue plasminogen activator (tPA), but tPA also increases the risk of hemorrhage, which is associated with BBB disruption [72, 73]. Stabilization of the BBB during and after ischemic stroke can improve the safety and efficacy of tPA treatment and reduce adverse outcomes. Combination therapy can provide additional benefits in some cases. Sac-1004, a BBB leakage blocker, may be used in combination therapy with tPA*.* As another point of view, in the present time, considering that only a small percentage of patients can receive tPA treatment, there is a need to develop new effective neuroprotective agents for the prevention and treatment of ischemic stroke, targeting mechanisms such as inflammation, oxidative stress, BBB disruption, excitotoxicity, apoptosis, and autophagy [4]. There is emerging interest in the study of neuroprotective compounds targeting more than one mechanism of ischemic stroke-related damage. Sac-1004 may be beneficial in the treatment of ischemic stroke through suppression of inflammatory responses and BBB disruption.

## Conclusions

In our present study, we demonstrate that the neuroprotective effects of Sac-1004 on I/R injury by the attenuation of BBB disruption and inflammatory responses. Sac-1004 could be therapeutically used for the treatment of ischemic stroke and other neurodegenerative diseases such as multiple sclerosis, vascular dementia, aging, and brain tumors related to BBB dysfunction.

## Additional file

Additional file 1: Figure S1. Sac-1004 increases HBMEC survival. HBMECs were starved and treated with various concentrations of Sac-1004. Chemical structure of Sac-1004 (A). Cell survival was detected using an MTT assay (B). Under the same experimental conditions, cell viability was also determined by microscopy after incubation for 48 h (C). All data are presented the means ± SEM. ****P* < 0.001. (JPG 82 kb)

## Abbreviations

BBB: Blood–brain barrier
GAPDH: Glyceraldehyde 3-phosphate dehydrogenase
GFAP: Glial fibrillary acidic protein
GLUT-1: Glucose transporter-1
HBMECs: Human brain microvascular endothelial cells
I/R: Ischemia-reperfusion
ICAM-1: Intercellular adhesion molecule-1
IL-1β: Interleukin-1 beta
IL-6: Interleukin-6
NF-κB: Nuclear factor-κB
PET: Positron-emission tomography
SMI-71: Sternberg Monoclonals Incorporated, product no. 71
TEER: Transendothelial electrical resistance
TNF-α: Tumor necrosis factor-alpha
tPA: Tissue plasminogen activator
VCAM-1: Vascular adhesion molecule-1
VEGF: Vascular endothelial growth factor
ZO: Zonula occluding
