# Supplementary figures and images for: Sac-1004, a vascular leakage blocker, reduces cerebral ischemia—reperfusion injury by suppressing blood–brain barrier disruption and inflammation
Source: J Neuroinflammation. 2017 Jun 23;14:122. doi: 10.1186/s12974-017-0897-3 (PMC5481915; doi:10.1186/s12974-017-0897-3)

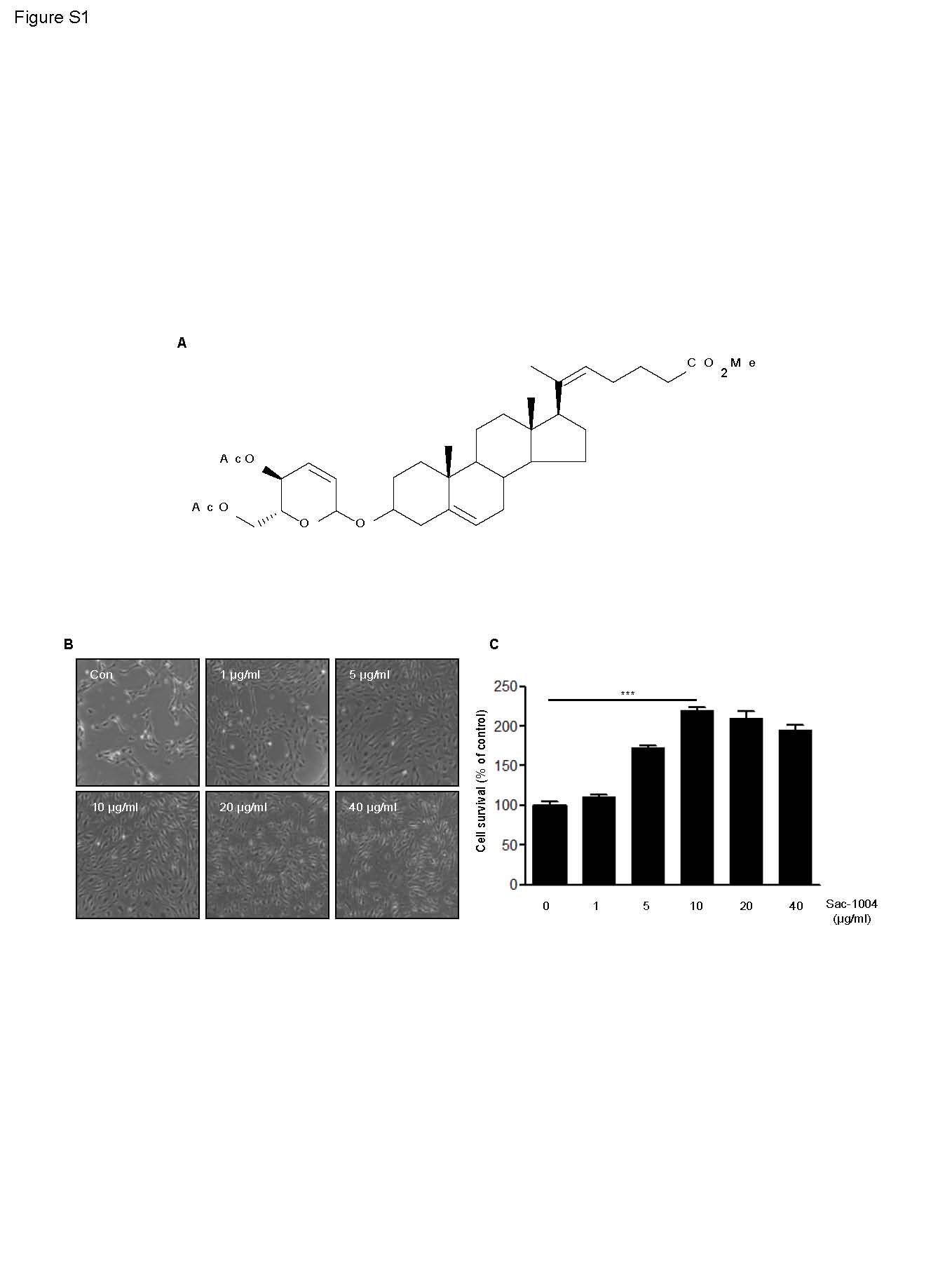

Supplement: Additional file 1: Figure S1. — Sac-1004 increases HBMEC survival. HBMECs were starved and treated with various concentrations of Sac-1004. Chemical structure of Sac-1004 (A). Cell survival was detected using an MTT assay (B). Under the same experimental conditions, cell viability was also determined by microscopy after incubation for 48 h (C). All data are presented the means ± SEM. ***P < 0.001. (JPG 82 kb) [file 12974_2017_897_MOESM1_ESM.jpg]
